# NAMPT, IL-6, and vaspin gene expressions and serum protein levels in type 2 diabetes mellitus and related complication

**DOI:** 10.55730/1300-0152.2688

**Published:** 2024-04-03

**Authors:** Süheyla Pınar ÇELİK, Damla Nur PARILTI, Leyla AÇIK, Mehmet Muhittin YALÇIN, İlhan YETKİN, Eldeniz YUNUSOV

**Affiliations:** 1 Department of Biology, Faculty of Science, Gazi University, Ankara, Turkiye; 2 Department of Endocrinology, Faculty of Medicine, Gazi University, Ankara, Turkiye

**Keywords:** Type 2 diabetes mellitus, NAMPT, IL-6, vaspin, adipokines, complications

## Abstract

**Background/aim:**

Type 2 diabetes mellitus (T2DM) is the most common type of diabetes and occurs due to insufficient insulin secretion or inability to use existing insulin and the effects of environmental factors. Although there are many studies on the pathophysiology of T2DM, the mechanisms contributing to the pathogenesis of insulin resistance and pancreatic beta-cell dysfunction have not been completely elucidated. Some adipokines secreted from adipose tissue, which are the primary regulators of insulin resistance, affect immune and inflammatory functions. Altered adipokine profiles have been observed in obesity and T2DM, leading to severe metabolic risks and changes in insulin sensitivity.

**Materials and methods:**

This study used quantitative PCR and ELISA techniques to analyze samples from individuals without diabetes (control group) and with T2DM (macrovascular and microvascular complications and without complications) for at least 10 years.

**Results:**

The mRNA expression and protein levels of NAMPT, IL-6, and vaspin genes were determined. While there was no significant difference in NAMPT, IL-6, and vaspin mRNA expression levels between diabetic groups, there was a significant decrease between the patient and control groups (p < 0.001). For serum protein levels, NAMPT protein levels decreased significantly in the uncomplicated group, while IL-6 and vaspin protein levels increased significantly in both microvascular and macrovascular complication groups (p < 0.001).

**Conclusion:**

The correlations between gene expressions, clinical parameters, and protein levels are crucial to understanding the implications of the findings.

## Graphical abstract

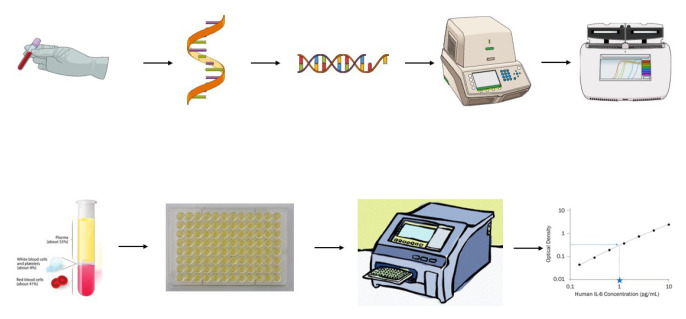

RNA was extracted from blood samples obtained from participants (three distinct groups of patients with type 2 diabetes and a healthy control group). cDNA was synthesized from the extracted RNAs, and the resulting data were analyzed by examining gene expression levels using qPCR. Serum samples collected from the same participants were analyzed by ELISA method, and serum protein levels were determined. Statistical analyses of gene expression data and serum protein levels were performed to evaluate whether there were significant differences between the groups.

## 1. Introduction

Diabetes mellitus (DM), caused by ineffectiveness or deficiency of the insulin hormone secreted by the beta islet cells of the pancreas, is affected by hereditary and environmental factors. It is a metabolic disease characterized by disorders in lipid, protein, and carbohydrate metabolism, accompanied by various complications and high blood glucose levels (fasting blood glucose ≥126 mg/dL, random plasma glucose ≥200 mg/dL, HbA1c ≥6.5%)^1^ (Dilworth et al., 2021). The most common classifications are type 1 diabetes mellitus (T1DM), type 2 diabetes mellitus (T2DM), gestational diabetes mellitus (GDM), and other types of diabetes. While T1DM is an autoimmune disorder resulting from the destruction of β-cells, T2DM is the most common type of DM due to insulin resistance, insulin secretion deficiency, and environmental factors. GDM happens during pregnancy and is not usually observed after pregnancy. In addition to other specific types of diabetes, there are various genetic defects, additional diseases, and the use of certain drugs (Dilworth et al., 2021).

T2DM is a disease that consumes necessary health resources, contributing to 8.4% of the deaths worldwide. Despite significant diagnostic and treatment advances, T2DM remains associated with increased mortality and morbidity compared to that of the general population (Nanayakkara et al., 2021). Diabetes is one of the top 10 causes of death worldwide. By 2021, Türkiye had the highest number and prevalence of diabetes in the International Diabetes Federation (IDF) European region. DM is one of the most common chronic metabolic diseases. According to the TURDEP II study data, the prevalence of diabetes was determined to be 16.5%, indicating that there are 6.5 million adults in Türkiye affected by this disease. The prevalence of diabetes is higher in women than in men (p = 0.008) (Satman et al., 2013; p = 0.008) in Türkiye. Diabetes, which was ranked 6th among the top 10 causes of death in Türkiye in 2000, rose to 5th place in 2019.^2^ According to IDF data, the diabetes-related death rate among individuals under the age of 60 years in Türkiye in 2021 was 4%. Approximately 83,220 people died of diabetes in 2021 (IDF, 2021).

In most cases, T2DM is associated with a variety of disabling and life-threatening complications, such as cardiovascular diseases, cerebrovascular diseases, nephropathy, and retinopathy (Rangel et al., 2019; Goyal et al., 2023). Although the exact causes of T2DM are not well understood, studies have shown that the development of T2DM is a multifactorial process involving a combination of genetic and nongenetic risk factors (Hansen, 2002). Studies have identified many risk factors for T2DM, such as ethnicity, family history of diabetes, age, sex, body mass index (BMI), obesity, sedentary lifestyle, overnutrition, low dietary fiber, central adiposity, alcohol, and smoking consumption, psychosocial stress factors, and environmental pollutants (Laakso, 2019; Abu-Shahba et al., 2021). These factors contribute to local or systemic low-grade chronic inflammation, which increases insulin resistance and leads to T2DM development (Abu-Shahba et al., 2021). Despite numerous studies that have been conducted on the pathophysiology of T2DM, the mechanisms underlying the pathogenesis of insulin resistance and pancreatic beta cell dysfunction still require further elucidation. In the context of T2DM, the identification of elevated circulating inflammatory factors such as cytokines, C-reactive protein (CRP), and chemokines in T2DM patients, as well as increased concentrations of some adipokines secreted by adipose tissue, associated with insulin resistance and pancreatic islet inflammation, creates a new field for understanding the pathophysiology, diagnosis, and treatment of T2DM (Cheng and Yu, 2022; Stanimirovic et al., 2022).

The cytokines secreted by adipose tissue are called adipokines, pivotal regulators of insulin resistance, and affect immune and inflammatory functions (Heo et al., 2019). Previous studies have evaluated the role of adipokines in the pathophysiological mechanism of T2DM and their potential as noninvasive biological candidates for managing T2DM, including diagnosis and treatment. As a typical example, patients with obesity and type 2 diabetes display altered adipokine profiles that lead to profound metabolic risks and changes in insulin sensitivity (Oh et al., 2016; Lee et al., 2019). Based on this, it has been hypothesized that therapeutically targeting inflammatory pathways may reduce the risk of T2DM or improve glycemic control in people with diabetes. NAMPT is an intracellular enzyme that plays an important role in ATP synthesis (Curat et al., 2006; Friebe et al., 2011). eNAMPT is secreted in many cell types, such as adipocytes and β cells, and functions as a proinflammatory cytokine in various signaling pathways such as IL-6/STAT3, PI3K/AKT. eNAMPT increases insulin secretion and protects from apoptosis in pancreatic β cells (Fukuhara et al., 2005). Interleukin-6 (IL-6) is a multifunctional cytokine secreted by many cell types. IL-6 also regulates and stimulates the release of chemotactic mediators, adhesion molecules, and acute phase proteins, leading to the release of other cytokines by enhancing the inflammatory response (Rodrigues et al., 2017). The role of IL-6 in type 2 diabetes and insulin resistance has not yet been clarified. While some studies have shown its negative effects on insulin (Klover et al., 2003; Lagathu et al., 2003), others have indicated that it is probably necessary to maintain glucose balance (Matthews et al., 2010; Wallenius et al., 2002). Vaspin (serpina12), isolated from adipose tissue of Otsuka Long-Evans Tokushima fatty (OLETF) rats, is an adipocytokine released from visceral adipose tissue. Vaspin, a serine protease inhibitor, is effective in insulin resistance, inflammation, and obesity (Hida et al., 2005). NAMPT, IL-6, and vaspin are significantly associated, potentiating each other’s effects. Based on the information mentioned above, we investigated NAMPT, IL-6, and vaspin gene expression, as well as serum protein levels, on T2DM development, diabetes complications, and the significant impact potential of these adipokines on T2D pathogenesis. We examined T2DM patients with micro- and macrovascular complications, those without complications, and a healthy control group living in Türkiye.

## 2. Method

### 2.1. Collection of type 2 diabetes and healthy control samples

We used data from the Gazi University Hospital Endocrinology and Metabolic Diseases Clinic, comprising individuals aged 18–70 diagnosed with T2DM for at least 10 years, categorized into groups with macrovascular complications (coronary artery disease, peripheral artery disease, and cerebrovascular diseases) (n = 40), microvascular complications (nephropathy, retinopathy, and neuropathy) (n = 40), and those without complications (n = 40), and healthy individuals (n = 40) aged 18–70 years with no known diabetes diagnosis. Blood and serum samples were collected. It was confirmed that the study participants had received COVID-19 vaccines and had not been diagnosed with COVID-19 until at least three months ago. Our study was approved by the Keçiören Training and Research Hospital Clinical Research Ethics Committee (decision no: 2012-KAEK-15/1873) and was conducted in accordance with the principles of the Declaration of Helsinki. Individuals who volunteered to donate blood were informed about the study, and their written and verbal consent was obtained.

### 2.2 NAMPT, IL-6, and vaspin gene expression analysis

The manufacturer’s protocol isolated total RNA using a HighPure RNA isolation kit (Roche Diagnostics, Mannheim, Germany). All samples were electrophoresed on a 1% agarose gel to assess total RNA integrity and visualized using a UV gel documentation system. Reverse transcription-polymerase chain reaction (RT-PCR) was performed using the Transcriptor First Strand cDNA Synthesis Kit (Roche Diagnostics) in a reaction volume of 20 μL according to the manufacturer’s instructions. The expression levels of target genes were analyzed using a RealTime PCR device (Roche Diagnostics, LightCycler 480 Instrument II) and LightCycler 480 Basic Software 1.5.1 (Roche Diagnostics). β-actin was used as a housekeeping gene. The RT-PCR mix consisted of 20 μL, comprising of 5 μL of cDNA product, 10 μL of 2X LightCycler 480 Probes Master Kit (Roche Diagnostics), 1 μL of TaqMan Gene Expression Assay (Roche Diagnostics) (Table 1), and 4 μL of water containing RNase (Roche Diagnostics). RT-PCR reactions were performed in triplicate, with a nontemplate negative control.

### 2.3. Investigation of NAMPT, IL-6, and vaspin protein levels

The manufacturer’s protocol was used to determine protein levels in serum samples using a Human ELISA Kit (Bioassay Technology Laboratory, Shanghai, China). The samples were centrifuged to observe the absence of precipitation in the serum samples. According to the kit protocol, 50 μL standards were prepared at five concentrations (320, 160, 80, 40, and 20 ng/L) using 640 ng/L of the original standard solution and standard diluent. A standard diluent was used as blank. Serum samples (40 μL) were collected, and 10 μL of the antibody was added. Blanks, standards, and samples were added to each well of a 96-well plate. Except for the blank well, 50 μL of streptavidin-HRP was added to the wells and incubated at 37 °C for 1 h. The whole plate was washed five times with 300 μL of wash buffer. Then, 50 μL of substrate A and 50 μL of substrate B were added to all wells and incubated at 37 °C for 10 min. Finally, 50 μL of stop solution was added, the plate was read in a plate reader at 450 nm.

### 2.4. Statistical analysis

When evaluating clinical features between type 2 diabetes cases and controls, the Tukey test was used after ANOVA to determine the difference between groups. Tamhane’s T2 test was used after Welch’s ANOVA. While calculating the statistics for NAMPT, IL-6, and vaspin plasma protein levels and NAMPT, IL-6, and vaspin gene expression levels between the groups, the normal distribution of the data was first checked. It was determined that the data did not show a normal distribution, and since there were more than two groups, the analysis was performed with the independent-samples Kruskal-Wallis test. This analysis was conducted using SPSS software (version 16.0; SPSS, Inc., Chicago, IL, USA). Differences were considered statistically significant at p ≤ 0.05. Significance values were adjusted for multiple testing using Bonferroni correction.

## 3. Results

Blood and serum samples were collected from 20 women and 20 men (mean age ± SD 62.30 ± 6.23 years) with macrovascular complications, 27 women and 13 men with microvascular complications (mean age ± SD 59.05 ± 6.83 years), and 26 women and 14 men without complications (mean age ± SD 58.65 ± 6.91 years). Controls were divided into four main groups comprising 26 females and 14 males (mean age ± SD 48.48 ± 7.93 years). NAMPT, IL-6, and vaspin expression levels were analyzed using RT-PCR, and serum protein levels were analyzed using ELISA. The relative quantification (RQ) of NAMPT, IL-6, and vaspin was calculated using the delta-delta Ct (ΔΔCt) method adjusted for the expression level of β-actin. The RQ value for the calibrator was equal to one (Livak and Schmittgen, 2001).

### 3.1. Clinical data

The clinical, biochemical, and genetic characteristics of the participants included in this study are summarized in Table 2. There was no significant difference between the diabetic and control groups in terms of sex (p > 0.05). The mean BMI of patients with T2DM without complications was 28.95 kg/m2, with microvascular complications was 29.16, with macrovascular complications was 29.84, and in the healthy control group, was 26.59 (interquartile range 40–52, n = 40). The mean duration of T2DM was 10 years. There was no significant difference between the diabetic and control groups in terms of sex, BMI, TG level, and T2DM duration (p > 0.05). However, a significant difference was observed between the diabetic patients and the control group regarding age, HbA1c, HDL-C, and LDL-C levels (p < 0.001). HbA1c values were significantly higher in the group with macrovascular complications (8.82%) than in the group without complications (7.84%). Additionally, HDL-C levels were considerably lower in the macrovascular complications group (43.05 mg\dL). LDL-C levels showed significant differences between the patient groups and control groups. They were found to be considerably lower in the macrovascular complication group (96.90 mg/dL) compared to the microvascular complication group (122.38 mg/dL) and the uncomplicated group (123.13 mg/dL). As expected, patients with diabetes had higher FPG levels than those in the control group (p < 0.001).

### 3.2. Gene expression analysis

**Figure 1:**
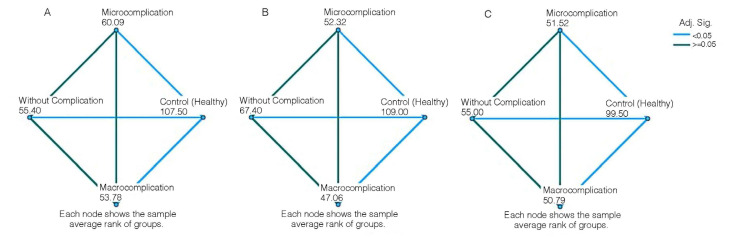
Statistical relationship between gene expression levels without complication, microvascular complications, macrovascular complications, and control groups (A: NAMPT gene expression, B: IL-6 gene expression, C: vaspin gene expression).

We found that NAMPT, IL-6, and vaspin mRNA expression significantly decreased at all investigated groups without complications (NAMPT: 0.55 ± 0.58 fold change, p < 0.001; IL-6: 0.51 ± 1.00 fold change, p < 0.001; vaspin: 0.70 ± 0.74 fold change, p < 0.001), with microvascular complications (NAMPT: 0.61 ± 0.62 fold change, p < 0.001; IL-6: 0.31 ± 0.65 fold change, p < 0.001; vaspin: 0.62 ± 0.70 fold change, p < 0.001), and with macrovascular complications (NAMPT: 0.56 ± 0.60 fold change, p < 0.001; IL-6: 0.33 ± 0.74 fold change, p < 0.001; vaspin: 0.85 ± 0.96 fold change, p < 0.001) (Table 2, Figure 1).

### 3.2. Analysis of serum protein levels

**Figure 2:**
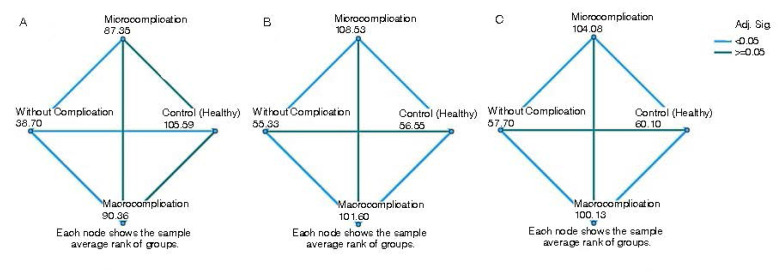
Statistical relationship between serum protein levels without complication, microvascular complications, macrovascular complications, and control groups (A: NAMPT protein level, B: IL-6 protein level, C: vaspin protein level).

NAMPT, IL-6, and vaspin protein levels in all investigated groups were 11.52 ± 4.49, 20.28 ± 5.23, 22.33 ± 5.30 in the group without complications, and 16.31 ± 4.81, 27.41 ± 6.81, 29.39 ± 7.88 in microvascular complications, 16.91 ± 5.90, 27.03 ± 7.97, 28.87±7.63 in macrovascular complications, and 17.47 ± 3.81, 20.28 ± 4.81, 22.36 ± 2.53 in the control group, respectively (Table 2, Figure 2). We found that NAMPT serum protein levels without complications were significantly lower than those in all other groups and the control groups. On the other hand, IL-6 and vaspin at micro/macrovascular complications were considerably higher than those in the groups without complications and the control group.

## 4. Discussion

Diabetes is a chronic and broad-spectrum metabolic disorder characterized by hyperglycemia, which occurs due to relative or absolute insulin deficiency or “insulin resistance” developed against the effect of insulin in peripheral tissues, affecting many organs and causing multisystemic involvement. T2DM accounts for 90–95% of all diabetes cases and mainly occurs after the age of 30. Patients are often obese or overweight. Exposure to chronic hyperglycemia has a significant impact on the quality of life and overall life expectancy by causing microvascular events such as nephropathy, retinopathy, neuropathy, as well as macrovascular events such as peripheral arterial disease, coronary artery disease, and stroke.

In our study, the patients were overweight or obese. Although the duration of T2DM was statistically similar, glucose and HbA1c levels were higher in the groups that developed complications. In T2DM, blood glucose, and HbA1c levels are used to evaluate diagnosis and treatment effectiveness. Additionally, their relationship with micro- and macrovascular complications has been confirmed by studies (Stratton et al., 2000; Seshasai et al., 2011).

Lipid abnormalities are also common in patients with diabetes and contribute to an increased risk of these complications. In our study, LDL-cholesterol, the treatment target, was similar or lower in the complication groups than in the other groups. This was due to more intensive cholesterol-lowering treatment of patients who developed complications.

In the present study, we report for the first time the role of NAMPT, IL-6, vaspin gene, and protein levels in Turkish T2DM patients with micro- and macrovascular complications, without complication, and a healthy control group. Furthermore, this is the first time that people with such long-term diabetes (10 years) have been studied in three separate complication groups. The results of our data specify that NAMPT, IL-6, and vaspin gene expression levels significantly decreased in all groups compared to control groups. The results of the present study indicate that NAMPT serum levels decrease considerably in the group without complications compared to all other groups and controls. Similarly, Alshahrani et al. (2019) stated that NAMPT expression is reduced in obese patients with T2DM, and metformin treatment reverts NAMPT expression to normal levels. However, Kieswich et al. (2016) showed that serum levels increased for monomeric eNAMPT in diabetic mice. In our study, protein levels and gene expression were lower in individuals with diabetes than in the controls. Previous studies have shown that these low levels may contribute to the development of cardiovascular complications (Hausenloy, 2009; Lovren et al., 2009; Naz et al., 2017). In a study by Catalán et al. (2011), the expression levels of NAMPT in blood monocytes were examined, revealing higher rates in obese individuals with T2DM compared to those with nonobese diabetes. The expression levels in nonobese individuals with T2DM were similar to those in controls (Catalán et al., 2011). Additionally, high gene expression was observed in studies involving obese individuals, and similar gene expression was noted in nonobese individuals and controls. This can be explained by the fact that the individuals participating in our study were not obese.

On the other hand, IL-6 and vaspin levels show a significant increase in micro/macrovascular complications compared to those in groups without complications and the control group. In another study, Jian et al. (2014) reported that obese diabetic patients had increased serum vaspin levels. Results from our research and other studies suggest that vaspin plays a role in human insulin resistance (Genc et al., 2011). It can inhibit a protease that plays a role in the degradation of a hormone with direct or indirect glucose and lipid-lowering effects (Genc et al., 2011). Vaspin gene expression has been shown to decrease with loss of body weight and progression of diabetes, and vaspin serum levels return to normal with insulin and pioglitazone treatment (Hida et al., 2005). In different studies, vaspin levels in individuals with diabetes have been found to be both lower (Jian et al., 2014; Sathyaseelan et al., 2016) and higher (Bilir et al., 2016; Montazerifar et al., 2018). Irregular production of IL-6 and long-term exposure lead to inflammation, which induces insulin resistance and overt T2DM. There is a mechanistic relationship between the stimulation of IL-6 and insulin resistance. IL-6 causes insulin resistance by impairing the phosphorylation of insulin receptors and receptor substrate-1, inducing the expression of SOCS-3, a potential inhibitor of insulin signaling (Rehman et al., 2017).

Elevated plasma levels of proinflammatory cytokines such as IL-6 were observed in T2DM patients compared to non-T2DM individuals (Kozakova et al., 2019; Randeria et al., 2019). According to the results of the metaanalysis study, T2D patients had high levels of IL-6, and the risk of developing T2D was increased in individuals with elevated IL-6 levels (Bowker et al., 2020).

NAMPT and vaspin play roles in the intricate molecular processes related to T2DM and obesity. Their involvement in metabolic regulation, inflammation, and insulin sensitivity highlights their potential significance as targets for therapeutic interventions or biomarkers for assessing metabolic health. However, the precise mechanisms and therapeutic implications of these proteins in T2DM and obesity are areas of ongoing research (Garten et al., 2015). In addition to NAMPT and vaspin, interleukin-6 (IL-6) is a well-known pleiotropic cytokine and adipokine that plays crucial roles in metabolism and immunity in various biological systems and organs (Kang et al., 2019).

Both genetic and environmental factors influence gene expression. Interactions between genetic variants and environmental influences may contribute to the observed differences in gene expression. Differences in the characteristics of study populations, such as age, sex, ethnicity, and lifestyle factors, can contribute to variability in gene expression. Furthermore, the statistical power of a study, influenced by factors such as sample size, can affect its ability to detect actual differences. In addition to that, IL6 expression can vary in different tissues. If the studies focused on other tissues or cell types, this could result in variations in the findings. Additionally, T2DM is a heterogeneous condition with various underlying mechanisms contributing to its development. Gene expression patterns may differ among subtypes of T2DM patients. Subgroup analyses based on disease duration, severity, or response to treatment might provide more insights.

Investigating the molecular mechanisms behind T2DM helps researchers and healthcare professionals better understand the underlying causes and pathophysiology of the disease. Molecular studies identify specific molecular pathways and targets that play a role in T2DM. This information is valuable for developing targeted therapies that can address the root causes of the disease, potentially leading to more effective and personalized treatments. Biomarkers are measurable indicators of a biological state and can be used for early diagnosis, prognosis, and disease monitoring. This capability can lead to earlier intervention and better management of the condition. Molecular insights into T2DM contribute to the development of new drugs and therapies. Researchers can design more effective drugs with fewer side effects by targeting specific molecules or pathways.

Upregulated adipokines in the development of obesity and T2DM generally have proinflammatory effects, leading to a chronic inflammatory state and contributing to metabolic dysfunction. In contrast, antiinflammatory adipokines benefit adiposity and insulin activity (Cheng and Yu, 2022). However, there are conflicting data regarding the circulating levels of some adipokines in T2DM. Therefore, instead of focusing on a single factor, studying the relationship between multiple adipokine balances and metabolic diseases would be more helpful in understanding the pathogenesis of T2DM. In this study, we investigated cytokines secreted by adipose tissue, which have promising functions in T2DM and obesity because they are key regulators of insulin resistance and affect immune and inflammatory processes.

This study determined that NAMPT, mRNA expression, and protein levels were lower than those of the control, and mRNA and protein results were consistent. In the case of IL-6 and vaspin, mRNA expression levels were lower; however, protein levels were higher. These contradictory results may be obtained because of the long mRNA half-life due to the drug treatments received by patients with diabetic complications. We conclude that IL-6 and vaspin mRNA half-life increased for unknown reasons, resulting in an unexpected increase in protein expression.

## Figures and Tables

**Table 1:** TaqMan Gene Expression Assays ID.

Name	Assay ID
TaqMan Gene Expression Assays, Gene NAMPT/Human	Hs00237184_m1
TaqMan Gene Expression Assays, Gene IL-6/Human	Hs00174131_m1
TaqMan Gene Expression Assays, Gene Vaspin/Human	Hs01100128_m1
TaqMan Expression Assays, Gene ACTβ/Human	Hs01060665_g1

**Table 2:** Clinical, biochemical, and genetic features of the subjects.

	Study group				
Characteristic	Without complication (n = 40)	Microvascular complication (n = 40)	Macrovascular complication (n = 40)	Healthy group (n = 40)	p
Sex (F:M)	14:26	13:27	20:20	14:26	0.290
**Age (years)**	**58.65 ± 6.91**	**59.05 ± 6.83**	**62.30 ± 6.23**	**48.48 ± 7.93**	**<0.001**
BMI (kg/m^2^)	28.95 ± 4.33	29.16 ± 5.74	29.84 ± 4.65	26.59 ± 3.62	0.073
T2D duration (years)	15 ± 5.11	16.05 ± 5.11	15.28 ± 5.69	-	0.658
**FBG (mg/dL)**	**157.4 ± 53.24**	**160.50 ± 63.33**	**176.58 ± 70.43**	**89.58 ± 8.14**	**<0.001**
**HbA** **_1c_** ** (%)**	**7.84 ± 1.90**	**8.20 ± 1.77**	**8.82 ± 1.81**	**5.75 ± 0.29**	**<0.001**
**HDL-C (mg/dL)**	**52.75 ± 13.83**	**51.68 ± 12.44**	**43.05 ± 8.27**	**54.60 ± 12.95**	**<0.001**
**LDL-C (mg/dL)**	**123.13 ± 79.26**	**122.38 ± 37.47**	**96.90 ± 34.81**	**145.85 ± 36.33**	**<0.001**
**T-C (mg/dL)**	**205.28 ± 44.67**	**205.43 ± 48.56**	**173.53 ± 43.55**	**225.85 ± 43.95**	**<0.001**
TG (mg/dL)	159.00 ± 79.26	160.23 ± 86.28	167.23 ± 84.36	127.13 ± 61.86	0.106
**Creatinine**	**0.71 ± 0.17**	**0.80 ± 0.32**	**0.89 ± 0.42**	**0.69 ± 0.17**	**0.011**
**NAMPT (ng/L)**	**11.52 ± 4.49**	**16.31 ± 4.81**	**16.91 ± 5.90**	**17.47 ± 3.81**	**<0.001**
**IL-6 (ng/L)**	**20.28 ± 5.23**	**27.41 ± 6.81**	**27.03 ± 7.97**	**20.28 ± 4.81**	**<0.001**
**Vaspin (ng/L)**	**22.33 ± 5.30**	**29.39 ± 7.88**	**28.87 ± 7.63**	**22.36 ± 2.53**	**<0.001**
***NAMPT***	**0.55 ± 0.58**	**0.61 ± 0.62**	**0.56 ± 0.60**		**<0.001**
***IL-6***	**0.51 ± 1.00**	**0.31 ± 0.65**	**0.33 ± 0.74**		**<0.001**
***Vaspin***	**0.70 ± 0.74**	**0.62 ± 0.70**	**0.85 ± 0.96**		**<0.001**

BMI, body mass index; FBG, fasting blood glucose; HbA1c, hemoglobin A1c; HDL-C, high-density lipoprotein cholesterol; LDL-C, low-density lipoprotein cholesterol; T-C, total cholesterol; TG, triglycerides; NAMPT, nicotinamide phosphoribosyltransferase; IL-6, interleukin-6.

## Footnotes

1 American Diabetes Association (ADA) (2017). Diagnosing diabetes and learning about prediabetes [online]. Website: http://www.diabetes.org/diabetes-basics/diagnosis/?loc=db-slabnav [accessed 14 March 2023].

2 World Health Organization (2020). The Global Health Observatory. Global health estimates: Leading causes of death [online]. Website: https://www.who.int/data/gho/data/themes/mortality-and-global-health-estimates/ghe-leading-causes-of-death [accessed 14 March 2023].

## References

[b1-tjb-48-02-133] Abu-Shahba N Mahmoud M El-Erian AM Husseiny MI Nour-Eldeen G 2021 Impact of type 2 diabetes mellitus on the immunoregulatory characteristics of adipose tissue-derived mesenchymal stem cells The International Journal of Biochemistry & Cell Biology 140 106072 10.1016/j.biocel.2021.106072 34455058

[b2-tjb-48-02-133] Alshahrani A AlDubayee M Zahra M Alsebayel FM Alammari N 2019 Differential expression of human N-alpha-acetyltransferase 40 (hNAA40), nicotinamide phosphoribosyltransferase (NAMPT) and sirtuin-1 (SIRT-1) pathway in obesity and T2DM: modulation by metformin and macronutrient intake Diabetes, Metabolic Syndrome and Obesity: Targets and Therapy 12 2765 2774 10.2147/DMSO.S228591 31920356 PMC6938199

[b3-tjb-48-02-133] Bilir BE Güldiken S Tunçbilek N Demir AM Polat A 2016 The effects of fat distribution and some adipokines on insulin resistance Endokrynologia Polska 67 3 277 282 10.5603/EP.a2016.0023 26884292

[b4-tjb-48-02-133] Bowker N Shah RL Sharp SJ Luan J Stewart ID 2020 Meta-analysis investigating the role of interleukin-6 mediated inflammation in type 2 diabetes EBioMedicine 61 103062 10.1016/j.ebiom.2020.103062 33096487 PMC7581887

[b5-tjb-48-02-133] Catalán V Gómez-Ambrosi J Rodríguez A Ramírez B Silva C 2011 Association of increased visfatin/PBEF/NAMPT circulating concentrations and gene expression levels in peripheral blood cells with lipid metabolism and fatty liver in human morbid obesity Nutrition, Metabolism, and Cardiovascular Diseases 21 4 245 253 10.1016/j.numecd.2009.09.008 20106640

[b6-tjb-48-02-133] Cheng JX Yu K 2022 New discovered adipokines associated with the pathogenesis of obesity and type 2 diabetes Diabetes, Metabolic Syndrome and Obesity: Targets and Therapy 15 2381 2389 10.2147/DMSO.S376163 35966830 PMC9371465

[b7-tjb-48-02-133] Curat CA Wegner V Sengenès C Miranville A Tonus C 2006 Macrophages in human visceral adipose tissue: increased accumulation in obesity and a source of resistin and visfatin Diabetologia 49 4 744 747 10.1007/s00125-006-0173-z 16496121

[b8-tjb-48-02-133] Dilworth L Facey A Omoruyi F 2021 Diabetes mellitus and its metabolic complications: the role of adipose tissues International Journal of Molecular Sciences 22 14 7644 10.3390/ijms22147644 34299261 PMC8305176

[b9-tjb-48-02-133] Friebe D Neef M Kratzsch J Erbs S Dittrich K 2011 Leucocytes are a major source of circulating nicotinamide phosphoribosyltransferase (NAMPT)/pre-B cell colony (PBEF)/visfatin linking obesity and inflammation in humans Diabetologia 54 5 1200 1211 10.1007/s00125-010-2042-z 21298414 PMC3071946

[b10-tjb-48-02-133] Fukuhara A Matsuda M Nishizawa M Segawa K Tanaka M 2005 Visfatin: a protein secreted by visceral fat that mimics the effects of insulin Science 307 5708 426 430 10.1126/science.1097243 15604363

[b11-tjb-48-02-133] Garten A Schuster S Penke M Gorski T de Giorgis T 2015 Physiological and pathophysiological roles of NAMPT and NAD metabolism Nature Reviews Endocrinology 11 9 535 546 10.1038/nrendo.2015.117 26215259

[b12-tjb-48-02-133] Genc H Dogru T Tapan S Kara M Ercin CN 2011 Circulating vaspin and its relationship with insulin sensitivity, adiponectin, and liver histology in subjects with non-alcoholic steatohepatitis Scandinavian Journal of Gastroenterology 46 11 1355 1361 10.3109/00365521.2011.603163 21770819

[b13-tjb-48-02-133] Goyal R Singhal M Jialal I Castano M 2023 Type 2 diabetes (nursing) StatPearls [Internet] Treasure Island, FL, USA StatPearls Publishing 33760496

[b14-tjb-48-02-133] Hansen T 2002 Type 2 diabetes mellitus--a multifactorial disease Annals of the Marie Curie-Sklodowska University. Section D: Medicine (English) 57 1 544 549 12898973

[b15-tjb-48-02-133] Hausenloy DJ 2009 Drug discovery possibilities from visfatin cardioprotection? Current Opinion in Pharmacology 9 2 202 207 10.1016/j.coph.2008.10.005 19081303

[b16-tjb-48-02-133] Heo YJ Choi SE Jeon JY Han SJ Kim DJ 2019 Visfatin induces inflammation and insulin resistance via the NF-κB and STAT3 signaling pathways in hepatocytes Journal of Diabetes Research 4021623 10.1155/2019/4021623 31396538 PMC6664505

[b17-tjb-48-02-133] Hida K Wada J Eguchi J Zhang H Baba M 2005 Visceral adipose tissue-derived serine protease inhibitor: a unique insulin-sensitizing adipocytokine in obesity Proceedings of the National Academy of Sciences 102 30 10610 10615 10.1073/pnas.0504703102 PMC1180799 16030142

[b18-tjb-48-02-133] International Diabetes Federation 2021 IDF Diabetes Atlas 10th edition Brussels, Belgium International Diabetes Federation

[b19-tjb-48-02-133] Jian W Peng W Xiao S Li H Jin J 2014 Role of serum vaspin in progression of type 2 diabetes: a 2-year cohort study PLoS ONE 9 4 e94763 10.1371/journal.pone.0094763 24732788 PMC3986225

[b20-tjb-48-02-133] Kang S Tanaka T Narazaki M Kishimoto T 2019 Targeting interleukin-6 signaling in clinic Immunity 50 4 1007 1023 10.1016/j.immuni.2019.03.026 30995492

[b21-tjb-48-02-133] Kieswich J Sayers SR Silvestre MF Harwood SM Yaqoob MM 2016 Monomeric eNAMPT in the development of experimental diabetes in mice: a potential target for type 2 diabetes treatment Diabetologia 59 11 2477 2486 10.1007/s00125-016-4076-3 27541013 PMC5506101

[b22-tjb-48-02-133] Klover PJ Zimmers TA Koniaris LG Mooney RA 2003 Chronic exposure to interleukin-6 causes hepatic insulin resistance in mice Diabetes 52 2784 2789 10.2337/diabetes.52.11.2784 14578297

[b23-tjb-48-02-133] Kozakova M Morizzo C Goncalves I Natali A Nilsson J 2019 Cardiovascular organ damage in type 2 diabetes mellitus: the role of lipids and inflammation Cardiovascular Diabetology 18 1 61 10.1186/s12933-019-0865-6 31077210 PMC6511166

[b24-tjb-48-02-133] Laakso M 2019 Biomarkers for type 2 diabetes Molecular Metabolism 27 Supplement S139 S146 10.1016/j.molmet.2019.06.016 PMC6768493 31500825

[b25-tjb-48-02-133] Lagathu C Bastard JP Auclair M Maachi M Capeau J 2003 Chronic interleukin-6 (IL-6) treatment increased IL-6 secretion and induced insulin resistance in adipocyte: prevention by rosiglitazone Biochemical and Biophysical Research Communications 311 2 372 379 10.1016/j.bbrc.2003.10.013 14592424

[b26-tjb-48-02-133] Lee MW Lee M Oh KJ 2019 Adipose tissue-derived signatures for obesity and type 2 diabetes: adipokines, batokines and micro RNAs Journal of Clinical Medicine 8 6 854 10.3390/jcm8060854 PMC6617388 31208019

[b27-tjb-48-02-133] Livak KJ Schmittgen TD 2001 Analysis of relative gene expression data using real-time quantitative PCR and the 2(-delta delta C(T)) method Methods 25 4 402 408 10.1006/meth.2001.1262 11846609

[b28-tjb-48-02-133] Lovren F Pan Y Shukla PC Quan A Teoh H 2009 Visfatin activates eNOS via Akt and MAP kinases and improves endothelial cell function and angiogenesis in vitro and in vivo: translational implications for atherosclerosis American Journal of Physiology-Endocrinology and Metabolism 296 6 E1440 E1449 10.1152/ajpendo.90780.2008 19351806

[b29-tjb-48-02-133] Matthews VB Allen TL Risis S Chan MHS Henstridge DC 2010 Interleukin-6-deficient mice develop hepatic inflammation and systemic insulin resistance Diabetologia 53 11 2431 2441 10.1007/s00125-010-1865-y 20697689

[b30-tjb-48-02-133] Montazerifar F Karajibani M Keikhaie MA Mohammadi M Jouy SH 2018 Serum adiponectin and vaspin levels in abdominal obesity and type 2 diabetes mellitus Iranian Journal of Diabetes and Obesity 10 1 23 30

[b31-tjb-48-02-133] Nanayakkara N Curtis AJ Heritier S Gadowski AM Pavkov ME 2021 Impact of age at type 2 diabetes mellitus diagnosis on mortality and vascular complications: systematic review and meta-analyses Diabetologia 64 2 275 287 10.1007/s00125-020-05319-w 33313987 PMC7801294

[b32-tjb-48-02-133] Naz S Sandhu QS Akhtar A Zafar U Khalid A 2017 Serum levels of visfatin and interleukin-6 in non-obese versus obese men with coronary artery disease Journal of the College of Physicians and Surgeons Pakistan 27 2 71 74 28292381

[b33-tjb-48-02-133] Oh KJ Lee DS Kim WK Han BS Lee SC 2016 Metabolic adaptation in obesity and type II diabetes: myokines, adipokines and hepatokines International Journal of Molecular Sciences 18 1 8 10.3390/ijms18010008 28025491 PMC5297643

[b34-tjb-48-02-133] Randeria SN Thomson GJA Nell TA Roberts T Pretorius E 2019 Inflammatory cytokines in type 2 diabetes mellitus as facilitators of hypercoagulation and abnormal clot formation Cardiovascular Diabetology 18 1 72 10.1186/s12933-019-0870-9 31164120 PMC6549308

[b35-tjb-48-02-133] Rangel ÉB Rodrigues CO de Sá JR 2019 Micro- and macrovascular complications in diabetes mellitus: preclinical and clinical studies Journal of Diabetes Research 2161085 10.1155/2019/2161085 30911551 PMC6397960

[b36-tjb-48-02-133] Rehman K Akash MSH Liaqat A Kamal S Qadir MI 2017 Role of interleukin-6 in development of insulin resistance and type 2 diabetes mellitus Critical Reviews in Eukaryotic Gene Expression 27 3 229 236 10.1615/CritRevEukaryotGeneExpr.2017019712 29199608

[b37-tjb-48-02-133] Rodrigues KF Pietrani NT Bosco AA Campos FMF Sandrim VC 2017 IL-6, TNF-α, and IL-10 levels/polymorphisms and their association with type 2 diabetes mellitus and obesity in Brazilian individuals Archives of Endocrinology and Metabolism 61 5 438 446 10.1590/2359-3997000000254 28225860 PMC10522244

[b38-tjb-48-02-133] Sathyaseelan AJ Adole PS Wyawahare M Saya RP 2016 Assessment of serum VASPIN levels among type 2 diabetes mellitus patients with or without acute coronary syndrome Journal of Clinical and Diagnostic Research 10 12 BC07 BC10 10.7860/JCDR/2016/22417.8952 PMC5296415 28208842

[b39-tjb-48-02-133] Satman I Omer B Tutuncu Y Kalaca S Gedik S 2013 Twelve-year trends in the prevalence and risk factors of diabetes and prediabetes in Turkish adults European Journal of Epidemiology 28 2 169 180 10.1007/s10654-013-9771-5 23407904 PMC3604592

[b40-tjb-48-02-133] Seshasai SRK Kaptoge S Thompson A Di Angelantonio E Gao P 2011 Diabetes mellitus, fasting glucose, and risk of cause-specific death The New England Journal of Medicine 364 9 829 841 10.1056/NEJMoa1008862 21366474 PMC4109980

[b41-tjb-48-02-133] Stanimirovic J Radovanovic J Banjac K Obradovic M Essack M 2022 Role of C-reactive protein in diabetic inflammation Mediators of Inflammation 3706508 10.1155/2022/3706508 35620114 PMC9129992

[b42-tjb-48-02-133] Stratton IM Adler AI Neil HA Matthews DR Manley SE 2000 Association of glycaemia with macrovascular and microvascular complications of type 2 diabetes (UKPDS 35): prospective observational study BMJ 321 7258 405 412 10.1136/bmj.321.7258.405 10938048 PMC27454

[b43-tjb-48-02-133] Wallenius V Wallenius K Ahrén B Rudling M Carlsten H 2002 Interleukin-6-deficient mice develop mature-onset obesity Nature Medicine 8 1 75 79 10.1038/nm0102-75 11786910

